# Young people's experiences of physical activity insecurity: a qualitative study highlighting intersectional disadvantage in the UK

**DOI:** 10.1186/s12889-024-18078-9

**Published:** 2024-03-15

**Authors:** Caroline Dodd-Reynolds, Naomi Griffin, Phillippa Kyle, Steph Scott, Hannah Fairbrother, Eleanor Holding, Mary Crowder, Nicholas Woodrow, Carolyn Summerbell

**Affiliations:** 1 Department of Sport and Exercise Sciences, Fuse, Durham University, Durham, DH1 3LA UK; 2 Population Health Sciences Institute, Fuse, Newcastle University, Newcastle, NE1 4LP UK; 3 Newcastle University Business School, Fuse, Newcastle University, Newcastle, NE1 4SE UK; 4 Health Sciences School, University of Sheffield, Sheffield, S10 2LA UK; 5 ScHARR, University of Sheffield, Sheffield, S1 4DA UK

**Keywords:** Physical activity insecurity, Adolescents, Young people, Disadvantage, Deprivation, LGBTQ +, Gender, Sexuality

## Abstract

**Background:**

Intersecting socioeconomic and demographic reasons for physical activity (PA) inequalities are not well understood for young people at risk of experiencing marginalisation and living with disadvantage. This study explored young people’s experiences of PA in their local area, and the associated impacts on opportunities for good physical and emotional health and wellbeing.

**Methods:**

Seven local youth groups were purposefully sampled from disadvantaged areas across urban, rural and coastal areas of England, including two that were specifically for LGBTQ + young people. Each group engaged in three interlinked focus groups which explored young people’s perceptions and lived experience of PA inequalities. Data were analysed using an inductive, reflexive thematic approach to allow for flexibility in coding.

**Results:**

Fifty five young people aged 12–21 years of different sexualities, gender and ethnicity took part. Analysis yielded four themes: PA experiences across spaces; resigned to a lack of inclusivity and ‘belonging’; safety first; complexities in access and accessibility. Young people felt more comfortable to be active in spaces that were simpler to navigate, particularly outdoor locations largely based in nature. In contrast, local gyms and sports clubs, and the school environment in general, were spoken about often in negative terms and as spaces where they experienced insecurity, unsafety or discomfort. It was common for these young people to feel excluded from PA, often linked to their gender and sexuality. Lived experiences or fears of being bullied and harassed in many activity spaces was a powerful message, but in contrast, young people perceived their local youth club as a safe space. Intersecting barriers related to deprivation, gender and sexuality, accessibility, disability, Covid-19, affordability, ethnicity, and proximity of social networks. A need emerged for safe spaces in which young people can come together, within the local community and choose to be active.

**Conclusions:**

The overarching concept of ‘physical activity insecurity’ emerged as a significant concern for the young people in this study. We posit that PA insecurity in this context can be described as a limited or restricted ability to be active, reinforced by worries and lived experiences of feeling uncomfortable, insecure, or unsafe.

## Background

Three in four adolescents do not meet global physical activity (PA) guidelines [1] and the annual global cost of inactivity is estimated to be in excess of $67·5 billion [2]. Adolescent inactivity is unequally distributed between nations, as well as within societies [3] and in England, only 47% of 13–16 year-olds met national PA guidelines in 2022/23 [4]. Physical activity is linked to 13 of the 2030 UN sustainable development goals (SDGs) including SDG3 good health and well-being, SDG4 quality education, and SDG10 reduced inequalities [1]. Through their global action plan, the World Health Organisation (WHO) [1] presents a mission to ensure access to safe and enabling environments along with diverse opportunities for PA, targeting a 15% relative reduction in inactivity for adults and adolescents by 2030. Despite this global focus, clear gaps in knowledge around policy development and implementation have been highlighted [3] with a need for supportive policies, environments, and opportunities [5] for children and young people to be active.

In this paper, we define physical activity as “people moving, acting and performing within culturally specific spaces and contexts, and influenced by a unique array of interests, emotions, ideas, instructions and relationships” ([6], p. 5). Like health, PA is heavily influenced by intersecting socioeconomic and demographic factors [7, 8], yet PA has the potential to improve health equity [9]. In England, epidemiological data show that children and young people are less likely to meet PA guidelines according to low affluence, gender (girls and 'other'), and ethnicity (Black, Asian, Mixed and Other non-white/non-white British) [4]. Evidence suggests, however, that individual determinants of young people’s PA are variable and diverse and include previous PA, PE/school sports, independent mobility and active transport, education level and other health behaviours such as alcohol consumption [10, 11]. A comprehensive systematic review of over 18-year-olds [12] reported 117 correlates of PA across a range of demographic, biological, psychological, behavioural, social and environmental factors.

The direct relationship between socioeconomic status (SES) and children’s PA is particularly unclear, with umbrella systematic review evidence [13] suggesting mixed findings in terms of whether SES is a determinant of PA, though the same study demonstrated a positive association between SES and PA for adults. Individual factors such as parental income and parental occupation, along with payment of fees/equipment did, however, show some evidence of an association with children and adolescent PA [13]. Whilst the authors note the small number of studies available for children and adolescents, a lack of causal evidence and differing measurement tools which might contribute to the uncertainty around SES and PA, we suggest also that quantitative evidence may well fail to capture the complexity of children and young people’s PA in different spaces. Indeed, a qualitative review of limited extant literature concerning socioeconomic position and experiences of barriers to PA [14] highlighted issues such as social support, accessibility and environment, and experiences (particularly gendered) of health and other behaviours, but importantly noted that those in low socioeconomic position areas had a good understanding of PA benefits. Better understanding is required regarding the complexity of PA experiences for children and young people living with disadvantage.

Within the PA literature, systems approaches are evolving to map and understand networks and mechanisms within complex systems, ultimately aiming to reduce health inequalities [15, 16], and a systems-based framework for action forms a key component of the WHO’s global strategy [1]. To support this work, better understanding is needed regarding the dynamic, contextual mechanisms which underpin various agents in local systems [17], for example through understanding better young people’s personal, or direct ‘lived experiences’ of PA. Engaging in dialogue with young people at the heart of local communities, offers a deeper and more nuanced understanding of place-based PA challenges and opportunities.

In general, individuals transitioning from childhood to adulthood are underserved in PA research, yet experiences earlier in life have a lasting effect on adult health and health behaviours [7]. The 2016 Lancet Commission on adolescent Health and Wellbeing [18] recommended setting clear objectives for change, based on local needs, and highlighted a gap for young people at risk of being socially and economically marginalised, including LGBT + (lesbian, gay, bisexual, trans and others) groups. Adolescents and those on the fringes of adulthood (hereafter referred to as young people) therefore present a critical but wide-ranging group with whom we must seek to better understand PA inequalities, particularly in the context of widening place-based inequality and deprivation and the syndemic 'shock' of the COVID-19 pandemic [19, 20]. Accordingly, we have applied the concept of intersectionality [21, 22] to explore the complex and intersecting factors which influence access to, and experiences of, PA.

We have recently reported young people’s nuanced understandings of the malleable and dynamic relationships between socioeconomic circumstance and health [23] and in this paper, we focused on PA specifically. We explored young people’s experiences of PA in their local area, and the associated impacts on opportunities for good physical and emotional health and wellbeing. In doing so we worked with young people who were already at risk of experiencing social and health inequalities across England, UK.

## Methods

### Overview

This paper drew on data from a larger project [23] where a series of three interlinked qualitative focus groups were undertaken with six groups of young people who attended local community youth groups between February and June 2021. For the present study, we recruited a further group (December 2021) to ensure diversity in terms of gender and sexual orientation. In total, 55 participants aged 12–21 years, from seven youth groups across three regions of England took part. Each youth group took part in three interlinked focus groups exploring health and health inequalities (21 focus groups in total). Two regions were in the north of England (South Yorkshire (SY) *n* = 2; North East (NE) *n* = 3; one region was in the south of England (London (L) *n* = 2). All regions fell within the most deprived quintile based on 2019 English indices of multiple deprivation (IMD) in England, with closer to 1 being more deprived. At participant-level, IMD quintile ranged from 1–3. The project commenced during the Covid-19 pandemic, where the UK experienced several lock-down periods. Due to social distancing restrictions, all focus groups were conducted online except for two youth groups which were in-person (one due to digital exclusion and another recruited once restrictions lifted sufficiently). Focus groups lasted approximately 1.5 h. Further details on methodological and ethical challenges and full procedures are described elsewhere [23, 24]. Ethical approval was granted by the School of Health and Related Research (ScHARR) Ethics Committee at the University of Sheffield and the Department of Sport and Exercise Sciences Ethics Committee at Durham University.

### Sampling

We adopted a purposive sampling strategy, designed to encapsulate maximum variation in perspectives and diversity [25]. Our sample was guided by the breadth and focus of the research question(s); demands placed on participants; depth of data likely to be generated; pragmatic constraints; and the analytic goals and purpose of the overall project [25, 26]. Our final sample included young people of different sexualities, gender and ethnicity across urban and rural and coastal areas (see Table 1).

**Table 1:** Demographic information

**Sample**	**Number of Participants**	**Age (years)**	**Gender**	**Ethnicity**	**Deprivation Position**^a^
Overall	55	Age range: 12–21 Average age: 16.5	19 Female 21 Male 4 Non-binary 9 Trans Male 1 Gender-Fluid 1 Trans masculine	41 White British 6 Asian/Asian British 3 Black/Black British 4 Mixed/Multiple ethnic group 1 Chinese	Average participant position = 9097 (Quintile 2)
South Yorkshire 1 (SY1)	6	Age range: 15–17 Average age: 15.5	3 Female 2 Male 1 Gender-Fluid	6 White British	Average participant position = 8009 (Quintile 2)
South Yorkshire 2 (SY2)	8	Age range: 13–17 Average age: 15.1	3 Female 5 Male	8 White British	Average participant position = 9414 (Quintile 2)
North East 1 (NE1)	7	Age range: 15–17 Average age: 15.8	2 Female 1 Male 2 Non-binary 2 Trans Male	7 White British	Average participant position = 15004 (Quintile 3)
North East 2 (NE2)	8	Age range: 13–20 Average age: 15.8	8 Male	8 White British	Average participant position = 1351 (Quintile 1)
North East 3 (NE3)	13	Age range:12–18 Average age: 14.3	2 Male 1 Female 2 Non-binary 7 Trans male 1 Trans masculine	11 White British 1 Mixed White/Asian 1 Mixed/other	Average participant position = 15,109 (Quintile 3)
London 1 (L1)	10	Age range: 16–21 Average age: 18.7	8 Female 2 Male	1 White British 5 Asian/Asian British 3 Black/Black British 1 Mixed/Multiple ethnic group	Average participant position = 7065 (Quintile 2)
London 2 (L2)	3	Age range: all aged 20 Average age: 20	2 Female 1 Male	1 Asian/Asian British 1 Mixed/Multiple ethnic group 1 Chinese	Average participant position = 7734 (Quintile 2)

^a^Deprivation position was calculated according to postcode in relation to 2019 English indices of deprivation

Youth workers invited group members to participate and shared an information video and project overview before researchers attended youth group sessions to discuss the study, build rapport and provide more detailed information sheets.These sessions were all held online during lockdown, except for two in-person groups, which were visited by the researchers. Written consent was gathered for all participants and, where under 16 years, opt-in consent from parents/guardians was also gained. Participants were asked to provide basic demographic information including postcode to calculate IMD.

### Data generation

Topic guides were developed [23], giving careful consideration to activities and language used around health inequalities. These were piloted and revised with two other partner youth organisations through early public involvement and engagement work. Youth workers helped facilitate sessions and at least four and two researchers were present for online and in-person sessions, respectively (NG, NW, MC, EH, HF, CDR, VE). The same groups of researchers worked across the 21 focus groups in different sites, to ensure consistency in process. All focus groups began with introductions and a warmup activity, followed by the main activity (in smaller breakout groups) and finally close and a cool-down activity. The three interlinked focus groups held with each youth group explored: (1) children and young people's understandings of health and wellbeing as a human right (via participatory concept mapping, see Jessiman [27] for an example), (2) children and young people's perceptions of the social determinants of health (sharing ideas about contemporary news articles relevant to health inequalities) and (3) children and young people's understandings of the ways young people can take action in their local area. Focus groups were recorded via encrypted Dictaphones and transcribed verbatim, with data anonymised at the point of transcription. Contextual field notes were taken by researchers.

### Analysis

Thematic analysis is a well-established approach to qualitative inquiry in health-related research that allows for the depth and richness of qualitative data to guide analysis [28]. We used an inductive, reflexive thematic approach to allow for flexibility in coding [26] and the desire to make sure our analysis was adequately capturing views of the young people themselves [29]. The approach was rigorously tested through the piloting of methods, regular analysis meetings, and sense-checking sessions (with participants) to validate themes [30]. For a full description of the original reflexive thematic analysis process [26, 31] please see Fairbrother et al. [23]. In brief, an initial coding frame was developed, with key codes and overarching themes discussed (linked to young people’s perspectives on the relationship between socioeconomic circumstances and health) and agreed upon by the wider research team. Once these core themes were established, an additional in-depth phase of reflexive analysis was undertaken (NH, PK, CDR, CS) to specifically explore PA, which had arisen continually, but not been developed as a theme, across the initial analysis. As before [23], we emphasised a creative and active approach to the analysis which followed an inherently ‘interpretative reflexive process’ ([26], p. 334). CDR, PK and NG were immersed in the data, continually reflecting upon, questioning and revisiting during the analysis process Regular analysis meetings took place to reflect and discuss and a new coding framework was developed and agreed by CDR, PK and NG, from which with themes were developed. The qualitative data management software system NVivo-12 was used to support data management.

## Results

Our analysis yielded four central themes: (1) PA experiences across spaces; (2) Resigned to a lack of inclusivity and ‘belonging’; (3) Safety first; (4) Complexities in access and accessibility. Nevertheless, themes naturally interrelate and the overarching concept of ‘PA insecurity’ emerged as a significant concern for the young people who generously shared their personal experiences with us. Here each interlinked focus group session is denoted S1, S2, S3.

### Physical activity experiences across spaces

The types of spaces in which young people felt able, or not able, to be active were crucial and formed the backdrop to their PA-related experiences and interactions with others. These are contextually linked here to later themes which provide further depth on how PA might or might not be enacted by young people within those spaces.

Across sites, there were differential responses in terms of ‘things to do’ in the local area. Inner city areas had fewer green and blue spaces but presented more organised opportunities in the locality. In rural areas young people had to travel to engage in social activities. Whilst in general there were positive attitudes towards PA, in the NE and SY, there was a perceived lack of things to do where they lived that did not cost money, or require private or unreliable public transport. A salient sub-theme developed around local opportunities for activity, with one group highlighting the resulting ease with which sedentary activities displaced other activities:

Facilitator*: ‘Do you prefer to play on consoles or do you prefer to go outside and run around and have exercise’?*

NE2, S2*: ‘If there’s nothing to do, then I will stay in the house, but if there is something to do, then I might as well just go outside’.*

At first glance, this apathy perhaps represents a lack of self-efficacy, often described as an individual-level determinant of PA. However, being physically active was far from simplistic and the young people described many associated challenges including closure of local amenities such as bowling and trampoline parks, with investment instead made in a nearby seaside town. For example, they described complexities around access to the nearest swimming pool. This was free in summer but not in the immediate locality, and thus required adult facilitation to enable the young people to travel to and access the pool, resulting in a structural barrier preventing them from taking part in something which was important to them within their existing social networks:

NE2,S2: ‘*Well just going out with friends and my dad saw that – I don’t know where – but he said, “Do you want to go?” “Yeah.” So he’ll get on the bus and he’ll go around and he got us in the baths*’.

NE2,S2: ‘*He goes around…and picks up children*’.

Spaces that were simpler to navigate included outdoor locations, largely based in nature, which for a number of the young people evoked a sense of freedom and well-being: ‘*there’s a big, massive field and a couple of times a week I take my dog there so he can meet other dogs. Take him for a big walk… is good for your health. It’s good for my dog*.’ (NE2, S1.)

Blue spaces were perceived similarly by those living near the coast: ‘*I like going to the beach… I just like the sea. It’s calm and obviously there’s a long way to walk as well*’ (NE2, S1).

Some indoor PA spaces, particularly swimming pools, were also described as places which evoked calmness and wellbeing. The following young person reflected on this in relation to how they felt in water:

‘*And it’s funny because when I first thought about the swimming pool, I didn’t think about it in terms of the physical exercise being good for me but obviously that is good. It’s much more that when I’m just completely submerged in water I feel very calm and I think it’s a bit of a shock to the system which can be nice, to be cold, suddenly very cold, and then get warmed up afterwards. So it’s kind of the pool and then also having a nice cup of tea when I get home after. For my mind and body I’d say…*’. (S1, S1).

Other indoor spaces such as gyms and sports clubs were spoken of in terms of being more for purposeful PA (i.e. exercise or sport) however the young people tended to speak less positively about their experiences, highlighting feelings of discomfort and of feeling self-conscious. In doing so, gender-based concerns often intersected: ‘*I did trampolining competitively…I was just getting to a point where I wasn’t comfortable. Because I was still having to wear the girl’s uniform …when you look at the differences between the uniforms, it's really stark*’. (NE1, S2). Similarly, in the gym setting, young people highlighted a perceived lack of security: ‘*Because gyms are enclosed spaces, there’s like dodgy blokes who are all like pumped and I’d rather not be around them. It’s just not my idea of fun*’ (SY2, S3). The ‘gym’ was repeatedly referred to in one focus group as negatively impacting upon self-esteem: ‘*I just hate it because like if you’re 16, the gym I used to go to had like a lot of older body building people so I’d feel like they were just watching me and I’d feel really uncomfortable about it’*, (SY1, S1). Conversations however also spoke of a need for inclusivity in the gym environment, noting feelings of pressure (as a female) *‘…if there’s guys looking at them they might not want them looking because obviously they’ll be looking in places they don’t want them looking. But also…we shouldn’t have to have girl-only gyms, everyone should be like integrated.*’ In the same conversation, concerns of racist behaviour were linked directly to the local area by another young person: ‘*And also racism as well, you could experience a lot of racism in gyms if you’re living in predominantly a white town.’* (SY2, S3).

The final space that permeated discussions, was an institutional one: the school or college environment. For the most part, narratives drew on personal experiences, often negative. For some, opportunities to be active in the institutional space had been removed altogether, something which was beyond their control: ‘*we didn’t do PE for about, a good three years because…we now needed to concentrate on our GCSEs…*’ (NE1,S3). Here, decisions made by adults in school, created barriers and the young people were aware of the gravity of lost opportunities to be active in the institutional space: ‘*…a lot of kids were missing out on that physical education and a way to exercise. That might have been students’ only way of exercise*’. This highlights how a lack of support from adults in positions of power can affect young people’s engagement in PA. Conversely, this could be positive intervention, illustrating how unequal distribution of support from teachers can impact significantly on young people’s PA experience in the education setting. In this example, one young person clearly highlighted a link between PA and psychological wellbeing:

‘*I managed to convince the teachers to let me do double rugby… two outside of school, one in school, like one club in school, two clubs outside of school and then two PE’s… because it’s an aggressive sport, I can get out all my aggression…I’m good with my team and I’m friends with all the people in it’*, (NE3, S3).

This theme illustrates that the young people were well aware of physical spaces in which they might be active but highlights the importance that young people attach to feelings of safety, security, and freedom, and how these can intersect with other characteristics. Physical activity spaces may be more conducive to positive mental health if they are larger and open, without interference from other people and where young people might feel less threatened by a perceived, or actual, need to conform.

### Resigned to a lack of inclusivity and ‘belonging’

For many of the young people, there was a commentary around feeling excluded from PA, particularly sport, linked to gender and sexuality. This appeared to evoke a sense of resignation, even at such a young age, of having had to give up trying to access certain types of PA due to feeling a lack of inclusivity.

‘*If you feel that you can’t participate in a sport, then your physical health is going to decline, just from the sake of a trans person just trying to negotiate – if you go to a game. Which dressing room are you going to use? You avoid that completely to keep yourself safe or you have then out yourself to people.*’ (NE1, S2).

Such experiences extended to PE lessons, sometimes with a sense of finality and relief, with one trans young person seemingly ‘owning’ that exclusion:

‘*Participant 2: I’m not doing P.E…I also have it on my notes saying that I can’t do PE after going through physiotherapy.*

*Facilitator 2: And is that good, do you think, because you don’t really want to do it?*

*Participant 2: Yeah.*’ (NE3, S3).

For some trans and non-binary young people who were engaging with PA at school, there appeared to be some support and understanding from staff, but this was not enough on its own: ‘*teachers keep coming and talking to me about joining in with the boys and I’ve finally got my mum to agree to let me go. But the teachers keep saying they’ll talk to her, but then my mum keeps saying she’ll ring the school but she never does*’ (NE3, S3). Another young person who had not ‘*come out’* as non-binary fully yet in school further illustrated negative experiences with gendered PE lessons: ‘*So I go into the girls’ PE and I’m sick of it because I go in and it’s just like, “hi girls!!” and I’m just like just kill me now*’. (NE3, S3).

For others, non-gendered opportunities in PE were desirable, avoiding traditional school curriculum activities that implied boys and girls taking part separately. One participant admitted to having hidden in the toilets to avoid PE because: ‘*I despise football but it was the only thing we did for about six months*’ and suggested a need for more ‘*variety*’ and ‘*more inclusive sports*.’ (NE1, S3).

### Safety first

Young people’s access to and engagement with PA in certain spaces, was foregrounded by a need to feel safe in those spaces both in terms of physical and emotional safety. For many this was linked to fear of crime and substance abuse in the local park: ‘*You could literally go…and it’s probably got either a bag that’s had something in it, alcohol bottles or needles. It’s quite terrifying*’ (SY1, S2). Others highlighted particular situations which required avoidance: ‘…*the dealing’s worse…that’s where all the fights happen…that basically…makes it more dangerous for people to be outside’* (L2, S2). When referring to the end of lockdown, and people buying drinking supermarket-bought alcohol ‘…*outside in the open, like, and in huge groups…’* one participant noted a fear for safety outdoors which intersected with worries of racist behaviour…’*so, like, that’s one of the things that makes me a little bit, like, scared, like, I wonder, like, would they, like, say something, like, racist to me?’* (SY1, S2).

Active travel was also explained as problematic, but for some a necessity which required precautions for ‘girls’ who it was suggested (by a group of boys) should ‘*Put a key in their fingers*’. (NE2, S2) and careful planning for one participant: ‘*I had to find a whole new route home so I didn’t get harassed and beat up*’. (NE3, S3.5). For one group, avoidance of crimes in progress was critical: ‘*people constantly starting fires*’ (NE2, S1). Fear of harassment outdoors seemed entrenched for many, sometimes linked to gender: ‘*Catcalling, and being followed… harder to feel safe… even in…broad daylight*’ (L2, S3), and other times a generational influence on feelings of fear in the local area: ‘…*after a certain time, 4 or 5 o’clock, my nan used to say “time to go back now” because she knew that that’s when all the dodgy people would come out really, even if it wasn’t necessarily dark earlier.*’ (SY1, S1).

For some, fears were more nuanced and centred around avoidance of bullying and transphobia: ‘*I think, particularly for trans and LGBTQ people, it’s difficult to feel secure in a sport … I don’t feel safe going to the club because if they find out I’m trans, they’ll just pick me out*’ (NE1, S2). This extended from access to sports clubs, to open spaces where young people in one LGBTQ + youth group were in agreement about fears of harassment based on their gender or sexuality:

*Facilitator 2: When you’re walking around and you’re out and about, how do you feel?*

*Participant 5: I feel like I’m in danger and scared….who feels unsafe out and about?*

*Participant 2: I do.*

*Participant 1: I do too.* (NE3, S3)

Together, these points exemplify a need for safe spaces in which young people at risk of marginalisation and living with deprivation can come together, connect within the local community and choose to be active: ‘*Like, because you can be at a park and then you can get harassed easily… You could barely go [to a park] without that [harassment] happening…Makes me not want to leave my house*.’ (NE1, S1). The youth groups themselves were seen as places of familiarity ‘*I’ve been here for ages*’, where young people can ‘*socialise, have fun, play a range of games, [make] new friends and bonding[sic]*’(NE2, S1) in a safe environment where social connections can be made. As such, youth groups might be pivotal in providing the kind of leadership and support required for PA access and engagement, perhaps even just in terms of open space provision: ‘*Even though we have the consoles, we don’t really use them that often. We’re mostly just outside…*’ (NE2, S1), as well as facilitating the social networks needed for young people to even consider PA.

### Complexities in access & accessibility

Whilst intersecting elements of PA access feature throughout other themes, it is important to draw specific attention to intersecting barriers relating to accessibility, including disability, Covid-19, affordability, and proximity of social networks with whom to engage in PA. In illustrating this, we draw on some of the issues highlighted in earlier themes.

Provision of physically accessible green spaces with appropriate facilities and equipment was an intersecting issue linked to crime and affordability, in the experience of one young person living in an area of poverty:

‘*…where I live and work, like football is huge. We've got a few football pitches, you’ve got to pay to play certain places especially for young people, so it's very, very difficult and what they tend to do, they’ll climb the cage gates and that leads to trouble and…stuff. If we had access to open free football pitches, that would be quite beneficial…I don’t think there’s…enough active stuff. Over the last couple of years, I've noticed they put in like monkey bars and other gym stuff [in parks].*’ (L1, S1).

In some discussions, disability was an intersecting issue, adding to the complexity of PA access and highlighting affordability and a desire to be active with peers:

*Participant 4: It’s not easy [to get about town with transport] if you have a hearing impairment and I know that from experience*

*Participant 1: But it can be easy though, depends on who your friends are, so me and my friends kind of live close [ind] and with covid, we have friends that kind of live further an it’s a little bit harder [ind]*

*Facilitator so why it is harder, what stops that*

*Participant 1: It’s probably either their parents not letting them go out further to come meet us [ind] and they may not be able to afford to…go out* (NE2, S1)

This theme illustrates that complexity of intersecting barriers to PA appears particularly pronounced and nuanced for some of these young people. As a result, solutions are likely to be similarly complex..

## Discussion

**Fig. 1:**
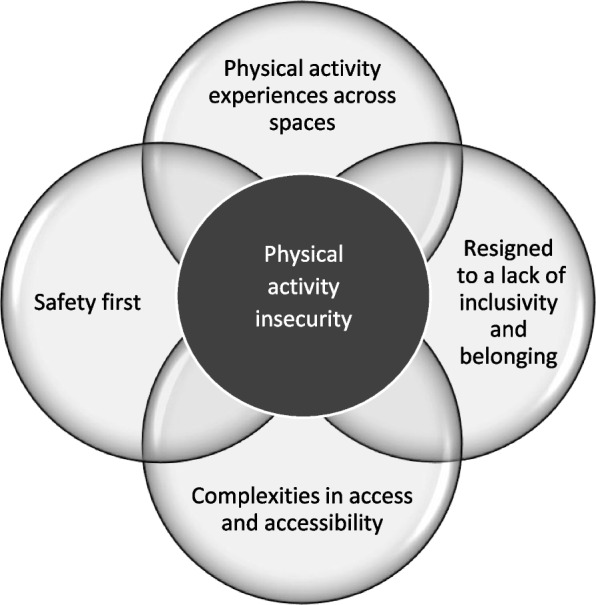
Physical activity insecurity experienced by young people

Considering the naturally intersecting themes in this study, we posit that the overarching concept of ‘physical activity insecurity’ emerged as a significant concern for the young people who generously shared their personal experiences (see Fig. 1). Physical activity insecurity is not an established term within the literature. To date and to the best of our knowledge, just one paper has linked it to families’ low readiness to *provide opportunities* for PA, where food insecurity was already being experienced: an “inability to provide sufficient health-promoting MVPA for children” ([32], p. 41). We note the distinction with food insecurity, which is well-recognised and formally defined as “limited or uncertain availability of nutritionally adequate and safe foods or limited or uncertain ability to acquire acceptable foods in socially acceptable ways.” ([33], p. 193). Here, we propose a new conceptualisation for PA insecurity, beyond simply providing a space for PA to be ‘secure’ and in recognition of the complexity of PA as a behaviour which is navigated cerebrally, socially, and politically within a situated space [6]. Young people in our study were very much aware of the spaces and opportunities for PA and associated potential benefits but were challenged by how the wider social and physical environment responded to them and reinforced feelings of inaccessibility. Here we draw on Friere’s concept of critical consciousness [34], which refers to an individual’s awareness of oppressive systemic forces in society, a sense of efficacy to work against oppression, to illustrate that instead of internalising the inaccessibility of certain spaces, young people actively highlighted the ways in which spaces were not set up with their access needs in mind. We thus define PA insecurity as a limited or restricted ability to be active, reinforced by worries and experiences of feeling uncomfortable, emotionally or physically unsafe. We suggest this can be as a result of oppressive practices, lack of inclusion and disadvantage. Our findings suggest that PA insecurity can be experienced by any young person at risk of experiencing marginalisation and living with disadvantage, particularly where intersectional barriers overlap. However it seems is particularly nuanced for transgender and non-binary young people, for example in dealing with harassment and/or exclusion due to gender discrimination. We suggest that the young people in our study may not ever be able to contemplate PA until they feel safer, supported and included by society. We explore this further, later on the discussion, in terms of existing theory related to feelings of oppression and discrimination in disablism [35, 36].

Challenges linked to gender and sexuality within a sporting context have been widely documented in the sociology literature, for example Anderson’s [37] inclusive masculinity theory suggests a trend towards reduced sexism and “homohysteria” in recent years. However, Pope [38] argues that though men’s attitudes to women in sport may be slowly changing for the better, overtly misogynistic masculinities are still prominent. Whilst our work was not grounded in theories of gender and sexuality, our sample comprised one trans masculine, one gender-fluid, four non-binary, nine trans males, 19 female and 21 male participants (Table 1) and our data certainly highlight that non cis-gendered individuals felt unable to be their true authentic self around PA. Little else is understood about the lived experiences of LGBTQ + youths in the PA domain, and what exists tends to consider school PE/sport provision [39]. Research does however support the notion that sexual and gender minority youths, particularly transgender young people, avoid PA settings due to feeling unsafe and uncomfortable [40, 41]. Herrick and Duncan [42] similarly highlight a need for safe, inclusive PA spaces for LGBTQ + adults, and also a need for an intersectional approach to explore PA complexity along with avoidance of elitist and inaccessible terms such ‘athlete’.

Intersectionality highlights the multiple intersecting identities of individuals and groups and how they interact and can compound each other in relation to oppression and inequality [21, 22]. In our findings, intersecting socioeconomic and demographic challenges raised by participants included deprivation (as per our sampling strategy), ablesim, crime and safety, affordability, and racism, as well as inequalities related to gender and sexuality. The young people in our study were cognisant to the ways in which different vulnerabilities can interact and compound each other, for example, exclusions related to homophobia, transphobia and ableism being further compounded by income inequality. They also discussed the links between accessibility and place, where some young people have greater opportunities to be involved in PA due to where they live, with closer proximity meaning greater access and affordability. Participants also reflected on how experiences of racism, sexism and/or homophobia in PA spaces increased the likelihood of disengagement. The young people who accessed LGBTQ + specific youth groups reflected on opportunities afforded to them to play or be active with others like them in a safe space, but highlighted that those space are not accessible to all, due to limited capacity and often a need to travel (affordability). Further insight into wider health inequalities as experienced by the LGBTQ + groups can be found in our linked paper by Griffin et al. [43]. In the present paper, we highlight a need to understand the complex ways in which intersectional disadvantages can intersect and compound each other, and in doing so, exacerbate PA insecurity.

We suggest that our findings of largely internalised feelings of insecurity, discomfort and a lack of safety represent facets of oppression and undermined psycho-emotional wellbeing. As such, there appear to be parallels with the concept of ‘psycho-emotional disablism and internalised oppression’ [35, 36, 44, 45] where “internalised oppression…can undermine someone’s psycho-emotional well-being and sense of self” ([44] p. 24). Reeve [44] further notes that the emphasis on removing psycho-emotional barriers should not lie with the individual, but rather with society. We posit that this has important implications for our findings. Reeve [45, 46] describes how indirect psycho-social disablism can reflect experiences of structural barriers, for example the “experience of being faced with an inaccessible building can evoke an emotional response such as anger or hurt at being excluded” ([46], p. 106). In our study such barriers are described by young people as e.g. changing rooms and uniforms which (drawing on Reeve [47]) alone might be characterised as solely socio-structural barriers to PA, if we did not have insight into how these experiences made the young people feel marginalised or resigned to inactivity. We suggest that the young people in our study similarly evoked elements of internalised oppression and discrimination in relation to PA, particularly in terms of feeling resigned to a lack of inclusivity and belonging. Importantly, we did not ask the young people in our study about their disability status, and therefore do not apply this theory through a disability lens per se. Rather, we consider here how psycho-emotional disablism might be applied through an intersectional lens, given the sharp similarities in challenges experienced and internalised by our young people. Given this, we suggest that simply adapting or removing structural barriers is insufficient to enable safe PA access for these young people, particularly those identifying as LGBTQ + . Researchers, practice partners and policy-makers need to work with young people to better understand their experiences, and to facilitate trustworthy relationships with PA within society.

Our findings also suggest that compassion, understanding and allyship of a trusted adult, may be critical for young people to feel safe and secure and thus give their trust and permission to engage in PA. Support from adults in positions of power had a strong influence on young people’s (lack of) engagement in PA, for example teachers in the institutional space. This point is supported by work which explored the relationship between a trusted adult and adolescent health and education outcomes [48], where young people outlined the need for mutual respect, patience and willingness of an adult to go the ‘extra mile’ in enabling them to engage in positive health behaviours. One US-based group has gone as far as to develop bespoke physical education teacher support around inclusive athletics for LGBTQ youth [49], though how this might work in practice across multiple PA settings needs yet to be explored. We suggest that young people, particularly those experiencing intersectional barriers to PA, should be included in decisions relating to PA policy and design of PA spaces themselves. This may in the longer-term reduce reliance on trusted adults.

Understanding what a secure PA space might actually look like for young people with shared challenges and/or protected characteristics is a clearly needed next step. Yet there exists a dearth of contextual evidence around how young people at risk of experiencing marginalisation and living with disadvantage experience PA in their local environment. More broadly, we acknowledge a need for whole system action to improve young people’s PA experiences in the spaces that they have access to. This extends beyond provision of what might be perceived physically safe spaces (e.g. safe playing, walking or cycling infrastructure) to inclusive language and action linked to changing facilities, clothing, and creating opportunities for PA within existing trusted networks such as youth groups. Though, as we have noted, responsibility for inclusivity should not lie with the individual, hearing the voices of young people in terms of who is needed, where they are needed and how spaces could be made more inclusive, is critical in this respect [50, 51].

In the UK, the PA landscape is driven by ‘top-down’ national policy agendas [52] and responsibility for young people’s PA provision is devolved across numerous sectors at local level. Given gaps in knowledge of PA policy development and implementation for young people [3] and the need for supportive policies, environments and opportunities to strengthen those national policy efforts [5] we suggest that further work might look to local groups and networks to co-produce, with young people at risk of marginalisation and living with disadvantage [53], guidance on what secure PA spaces might look like and who is required to facilitate them. Such work must carefully consider the views of young people, trusted adults (e.g. youth workers) and others involved in provision of PA such as service providers, teachers and local authorities.

### Limitations

Fieldwork took place during periods of Covid-related lockdown, and some comments from participants may reflect challenges which were exacerbated at this point in time. Though there was ethnic diversity across the overall sample, this was largely limited to the southern sites. We also acknowledge potential limitations of recruitment through existing youth organisations, which may exclude the voices of young people who are unable to engage with this provision. Nevertheless, as noted by Fairbrother et al. [23], working with youth groups enabled us to have the support of Youth Workers in refining topic guides and facilitating participant engagement, as well as providing an invaluable source of trusted support for participants [24].

### Strengths

Young people were recruited through youth groups and trusted youth leaders were very much part of the process. In each sampling site, the same group of young people engaged in three focus groups, and the building of rapport through this process provided open and honest reflections. We note the rigour of analysis as a strength in this work, particularly the sense-checking of themes with the young people.

### Future research

Future research should build on these findings and work with young people at risk of experiencing marginalisation and living with disadvantage to explore what safe PA spaces and associated PA policies might consist of. Further diversity in sampling is also important. Finally, consideration should be paid to whether PA insecurity can be measured, for example via an assessment tool.

## Conclusions

We argue that the voices of young people at risk of marginalisation and living with deprivation, including LGBTQ + youths, must be heard in the context of their own embodied PA experiences, in order to mediate PA inequalities. Young people articulated a clear and in-depth understanding of the spaces in which they experience (or do not) PA. They provided a powerful narrative which suggests PA insecurity as central to their lived experiences of PA, often highlighting intersecting barriers to PA which resulted in feelings of internalised oppression and undermined psycho-emotional well-being. We highlight a need for accessible and affordable safe spaces within the local community, where young people can come together and have the ability to be active. Such safe spaces will likely require facilitation and support of trusted adults in terms of helping to manage the complexity of challenges associated with PA for these young people.

## Data Availability

The datasets generated and analysed during the current study are not publicly available due to privacy reasons but are available from the corresponding author on reasonable request.
